# Editorial: Ongoing advancements in the research of radiation- induced toxicity and the development of interventions to protect and/or mitigate its effects

**DOI:** 10.3389/fpubh.2024.1471228

**Published:** 2024-08-21

**Authors:** Wanchang Cui, P. Artur Plett, Guru Prasad Sharma, Sarah Kerns, Thomas J. MacVittie

**Affiliations:** ^1^Armed Forces Radiobiology Research Institute, Uniformed Services University of the Health Sciences, Bethesda, MD, United States; ^2^The Henry M. Jackson Foundation for the Advancement of Military Medicine, Inc., Bethesda, MD, United States; ^3^Department of Pharmacology and Molecular Therapeutics, Uniformed Services University of the Health Sciences, Bethesda, MD, United States; ^4^Department of Medicine, Indiana University School of Medicine, Indianapolis, IN, United States; ^5^Department of Radiation Oncology, Medical College of Wisconsin, Milwaukee, WI, United States; ^6^Department of Radiation Oncology, University of Maryland School of Medicine, Baltimore, MD, United States

**Keywords:** radiation, partial body irradiation, countermeasure, sex, polypharmacy, nuclear evacuee

Radiation-induced toxicity is a complex dose- and time-dependent multiorgan effect. Current research has made progress in addressing radiation-induced toxicity, as evidenced by the FDA approval of eight medical countermeasures (MCMs) against the lethal effects of hematopoietic acute radiation syndrome (H-ARS); four primary MCMs were initially approved, followed by four biosimilars, all focused on mitigating the myelosuppressive and potentially lethal effects of H-ARS (https://www.fda.gov/emergency-preparedness-and-response/mcm-issues/radiological-and-nuclear-emergency-preparedness-information-fda). However, important questions remain; there are no post-exposure MCMs against the lethal gastrointestinal ARS (GI-ARS) nor against the delayed effects of acute radiation exposure (DEARE) or the effects of polypharmacy on multiorgan injury in survivors, which impacts the hematopoietic system and organs affected by the high-threshold effect characteristics of the DEARE. MCM devlopment against multiorgan injuries associated with DEARE requires validated animal models that accurately represent organ-specific injuries in survivors of acute ARS. Additionally, it is crucial to determine if acute and delayed radiation sequelae and respective treatment(s) are sex-dependent. Furthermore, do radiation-related events cause psychological stress in vulnerable individuals? To address these concerns, the latest research on acute radiation exposure and its biological effects has been compiled into the current Research Topic in *Frontiers in Public Health*.

The Research Topic, featuring five research articles by leading authors from universities and institutes around the world, covers a wide range of studies related to the effects of acute radiation exposure on human health. The covered topics include mechanisms of radiation-induced toxicity, establishing a realistic radiation-effect animal model, the pharmacological interventions to mitigate radiation toxicity and the potential sex-dependent effects, and finally a case report about the stress of a vulnerable evacuee following a nuclear power plant accident. Specifically, Kiang et al., demonstrated that combined therapy with ciprofloxacin and pegylated G-CSF can mitigate brain hemorrhage and reduce mortality following high-dose irradiation, highlighting the potential of combination therapies to address complex multi-organ radiation effects. A related study by Horseman et al., studied the effects of combined ciprofloxacin and pegylated G-CSF therapy on the intestinal pathology and gut microbiota changes, offering insights into multi-faceted approaches to mitigate gastrointestinal toxicity. Beach et al., established a partial body irradiation model with ~2.5% bone marrow shielding in C57L/J mice to study the delayed effects of acute radiation exposure, providing a framework for understanding long-term toxicity and refining predictive models. Xiao et al., studied the sex-specific effects of an amino acid-based radiation mitigator, underscoring the importance of personalized approaches based on biological differences. A case report by Yamamura et al., highlighted that evacuation following the Fukushima Daiichi nuclear accident may have induced significant stress to a vulnerable patient, pointing to the need for holistic approaches that address both physical and psychological health.

Overall, this Research Topic provides readers a glimpse into the latest advancements in radiation research, the mechanisms of radiation toxicity, and the ongoing need for research into post-exposure mitigation strategies. Featuring research articles from leading experts in the field, this Research Topic is an essential read for anyone interested in the impact of acute radiation exposure on human health.

## Author disclaimer

The views and opinions expressed here are those of the authors and do not necessarily reflect the views or positions of any entities they may represent. These entities may include the Armed Forces Radiobiology Research Institute (AFRRI), the Uniformed Services University of the Health Sciences (USUHS), the Henry M. Jackson Foundation for the Advancement of Military Medicine, Inc. (HJF) or the Department of Defense (DoD), Indiana University, the Medical College of Wisconsin, and University of Maryland, School of Medicine.

